# Complement C1q as a Potential Biomarker for Obesity and Metabolic Syndrome in Chinese Adolescents

**DOI:** 10.3389/fendo.2020.586440

**Published:** 2020-11-30

**Authors:** Xuelian Yang, Yanan Ma, Zhongyi Zhao, Shihan Zhen, Deliang Wen

**Affiliations:** ^1^ Institute of Health Sciences, China Medical University, Shenyang, China; ^2^ School of Public Health, China Medical University, Shenyang, China; ^3^ Department of Health Management, Shengjing Hospital of China Medical University, Shenyang, China

**Keywords:** C1q, biomarker, adolescent, obesity, metabolic syndrome

## Abstract

**Background:**

Complement C1q (C1q) has been confirmed to be related to obesity, metabolic syndrome (MetS), and its components. However, human data regarding the associations are relatively scarce. This study aimed to investigate associations of C1q with obesity as well as MetS in Chinese adolescents.

**Methods:**

A total of 1,191 Chinese adolescents aged 13–18 years were enrolled in this study. The biochemical and anthropometric variables of all the subjects were evaluated using standardized procedures. C1q was measured using the immunoturbidometric assay. The relationship between C1q and obesity or MetS was analyzed using multiple regression analyses.

**Results:**

Obesity was more prevalent among participants in the highest tertile than in the lowest tertile of C1q levels. The highest tertile of C1q was related to a greater effect on the risk of MetS, and its trend test was statistically significant. Except for hyperglycemia, the prevalence of other components of MetS significantly increased relative to an increase in C1q tertile. Receiver operating characteristic (ROC) curve analysis of C1q for predicting adolescents with MetS illustrated that the area under the curve (AUC) was 0.82 [95% confidence interval (CI): 0.76, 0.88; *P*<0.001] in the total population after adjusting for confounders.

**Conclusions:**

This study observed a significantly higher prevalence of obesity and MetS features in adolescents with high C1q. The findings of the current study also reported a significant relationship between C1q levels and MetS components [except for fasting plasma glucose (FPG)] in Chinese adolescents. C1q may represent a biomarker for predicting obesity or MetS in adolescents.

## Introduction

As a major challenge for public health (1), the upward trend of childhood overweight and obesity is generating direct and indirect costs, including lifetime healthcare and productivity costs (2). Childhood obesity has also been associated with the risk of metabolic syndrome (MetS) (3). As previously reported, MetS is a cluster of cardiovascular risk factors, including central obesity, hypertension, hyperglycemia, hypertriglyceridemia, and low levels of high-density lipoprotein cholesterol (HDL-C) (4–6). In addition, MetS was found to influence the risk of cardiovascular diseases, type II diabetes mellitus, chronic kidney disease, non-alcoholic fatty liver disease, and all-cause mortality (7–11). Nowadays, for children and adolescents with MetS, prevalence rates in both developed and developing countries are increasing (6, 12, 13). Considering the scale and problems of obesity and MetS among adolescents, as well as significant and sustained adverse effects on health, it would be critical to identify novel biomarkers for predicting obesity and MetS which would then play a significant role in prevention.

As an important part of the innate immune system (14), the complement system was associated with several components of MetS, including obesity and insulin resistance (15, 16). In adipose tissues of people with obesity, there have been increased expressions of specific complement components (17). Complement C1q (C1q), widely produced by macrophages, immature dendritic, and mast cells (18–23), was referred to as a pattern recognition receptor of the innate immune complement system (24). It is a protein consisting of 18 polypeptides chains of three different types named A, B, and C (25). Recently, these three C1q sub-chains were demonstrated to be upregulated among people with obesity aged 22–36 years (26). Accumulating evidence has indicated that C1q was significantly associated with both cardiovascular disorders, including arterial stiffness, and metabolic health outcomes, such as diabetes mellitus (27–29). In alcoholic liver disease models, C1q was considered to be a key mediator of adipose inflammation caused by alcohol exposure (30–32). According to studies on the complement system and MetS, focus has mostly been on serum complement C3 and its effects on the risk of MetS in both Chinese and Caucasian subjects (33, 34); few studies have addressed the relationship of C1q and MetS. Based on a cross-sectional research among 127 Japanese individuals, serum C1q level was confirmed to be positively associated with blood pressure (27), which has been referred to as a component of MetS (5).

As it stands, most available studies on the relationship between C1q and obesity have mainly been conducted in adults, whereas data on adolescents is largely lacking. With respect to C1q and MetS, previous studies were usually concerned with single or partial components of MetS. Moreover, whether serum C1q level could be beneficial to human subjects with MetS is not yet well-understood, especially in adolescents. In the present study, our objectives were to determine the significance of C1q in adolescents with obesity and to investigate the association of C1q with MetS and its components.

## Materials and Methods

### Study Population

Data of the current study were extracted from the 2017 to 2018 Huanggu District Middle and Primary School Student Physical Fitness Monitoring (HMPSPM) database. The study was a cross-sectional observational study of adolescents enrolled in five middle schools during the 2017–2018 school year in Huanggu District, Shenyang, China. Individuals with missing information, older than 18 years, and with psychiatric disease or severe systemic disease were excluded from this study. In total, 1,191 adolescents aged 13–18 years were included in the analysis. The study was approved by the China Medical University Health Science Ethics Committee, and was conducted in accordance with the principles of the Declaration of Helsinki. Written informed consent of all the participants and/or their parents were obtained before entering the study.

### Anthropometric and Biochemical Measurements

As previously reported (35), all subjects were measured between 8 am and 10 am, and the anthropometric measurements were conducted by a trained research assistant who followed reference protocols recommended by the World Health Organization (WHO) (36). The researcher used standardized equipment to measure the weight and height of each participant to the nearest 0.1 kg and 0.1 cm, respectively. Participants were instructed to wear light clothing and were measured barefoot. Weight was divided by height squared to obtain body mass index (BMI) (kg/m^2^). In light of Chinese reference values, age- and sex-speciﬁc Z-scores of BMI were calculated for all the participants. Waist circumference (WC) was taken for all participants using established techniques (36). A standardized Omron i-C10 blood pressure monitor (Omron Healthcare Co., Ltd, Kyoto, Japan) was used to measure sitting blood pressure.

Venous blood samples were collected by nurses after participants had fasted overnight for about 12 h. All the samples were immediately transported to the Department of Clinical Laboratory, Shengjing Hospital of China Medical University, where the following parameters were measured by use of standard operating procedures. Levels of alanine aminotransferase (ALT), aspartate aminotransferase (AST), alkaline phosphatase (ALP), and gamma glutamyl transpeptidase (GGT) were determined with an International Federation of Clinical Chemistry method. HDL-C and low-density lipoprotein cholesterol (LDL-C) were assayed directly using the selective solubilization method, and a novel homogeneous enzymatic assay was used to determine small dense low-density lipoprotein cholesterol (sdLDL-C) levels. Concentrations of total triglyceride (TG) were measured by the standard enzymatic method. For fasting plasma glucose (FPG), a modified hexokinase enzymatic method was used, and apolipoprotein A1 (ApoA1), apolipoprotein B (ApoB), and C1q were measured by immunoturbidometric assay.

### Determination of Overweight and Obesity

To define overweight and obesity in adolescents, categorization was based on criteria set by the WHO. Overweight was defined as BMI-for-age greater than one standard deviation above the WHO Growth Reference median, and obesity was defined as greater than two standard deviations (1).

### Definition of Metabolic Syndrome

MetS and its components in the present study were defined by the criteria of MetS developed by the International Diabetes Federation (IDF) (37). MetS was identified when abdominal obesity (defined as WC≥ 90th percentile for age and gender for individuals between 10 to 16 ages and WC≥90 cm for males or ≥80 cm for females in adolescents over 16 years old) and the following two or more criteria existed simultaneously: 1) high blood pressure (systolic blood pressure≥130mmHg or diastolic blood pressure≥85mmHg); 2) elevated plasma glucose (FPG≥5.6mmol/L); 3) elevated TG (TG≥1.7mmol/L); 4) low HDL-C (HDL-C<1.03mmol/L in both sexes aged 10 to 16 years and <1.03mmol/L for males or <1.29mmol/L for females in adolescents over 16 years old).

### Statistical Analysis

Skewness and kurtosis tests were performed for the normal distribution of the data. Descriptive information was presented as means and standard deviations for Gaussian distributions and as medians, together with the upper and lower quartiles, for non-Gaussian distributions. For categorical variables, numbers and percentages were reported. Concentrations of C1q were divided into tertiles. Characteristics of the participants were presented according to the C1q tertiles. Youden’s index, a measure of overall diagnostic effectiveness, was used to investigate a C1q cut-off in predicting MetS. Based on the optimal cut-off value of C1q, subjects were divided into “lower” and “upper” groups. Multiple linear regression was performed to identify the association between C1q and BMI Z-scores. Adjusted odds ratios (ORs) and 95% confidence intervals (CIs) were calculated using multiple logistic regression analysis to assess the relationships between C1q and overweight/obesity, obesity, MetS, and MetS components according to C1q tertile. C1q tertile1 was set as the reference. The first multiple regression analysis models adjusted only for age, whereas subsequent models adjusted for age in addition to other potential confounding factors. The receiver operating characteristic (ROC) curve analysis was used to explore sensitivity and specificity. The area under the curve (AUC), together with 95% CI, was used to determine whether C1q could be a biomarker for predicting adolescents with MetS. Data were analyzed using Stata (Version 15.1; StataCorp, College Station, TX, USA), and *P* values were reported as two-tailed with *P* < 0.05 indicating statistical significance.

## Results

### Characteristics of the Study Population

The basic anthropometric and clinical information of the 1,191 subjects aged 13–18 years, stratified by C1q tertiles, are shown in **Table 1**. In the third C1q tertile, the proportion of overweight, obesity, MetS, and MetS components (including central obesity, hyperglycemia, high TG, and low HDL-C) were highest. The optimal cut-off value for C1q was 184.7mg/L and Youden’s index was 0.30. Results after classifying C1q according to the cut-off value are shown in **Supplementary Table 1**.

**Table 1:** Characteristics of the study population according to tertiles of C1q.

Characteristic	Tertile1 (n=397)	Tertile2 (n=397)	Tertile3 (n=397)
Age, mean (SD), years	16.25 (0.98)	16.25 (0.99)	16.16 (1.02)
Boys, No. (%)	295 (74.31)	194(48.87)	89 (22.42)
Anthropometry			
BMI z-score, mean (SD)	-0.25 (0.75)	0.09 (1.03)	0.24 (1.09)
Weight status, No. (%)			
NW	323 (81.36)	268 (67.51)	241 (60.71)
OW	49 (12.34)	75 (18.89)	85 (21.41)
OB	25 (6.30)	54 (13.60)	71 (17.88)
Waist circumference, mean (SD), cm	72.39 (8.56)	74.88 (11.49)	75.27 (12.04)
Metabolic syndrome outcomes, No. (%)			
Metabolic syndrome	6 (1.51)	25 (6.30)	29 (7.30)
Central obesity	40 (10.08)	81 (20.40)	115 (28.97)
Hypertension	79(19.90)	98 (24.69)	93 (23.43)
Hyperglycemia	2 (0.50)	4 (1.01)	8 (2.02)
High TG	13 (3.27)	17 (4.28)	29 (7.30)
Low HDL-C	71 (17.88)	106 (26.70)	130 (32.75)
Laboratory examinations, median (Q1, Q3),			
ALT, (U/L)	26 (11, 64)	30 (11, 62)	29 (12, 62)
AST, (U/L)	15 (13, 18)	16 (13, 18)	15 (13, 18)
ALP, (U/L)	103 (79, 137)	96 (75, 121)	87 (75, 109)
GGT, (U/L)	16 (13, 19)	16 (12, 22)	16 (13, 22)
FPG, (mmol/L)	4.22 (3.94, 4.52)	4.25 (3.98, 4.53)	4.38 (4.09, 4.67)
HDL-C, (mmol/L)	1.31 (1.13, 1.52)	1.27 (1.14, 1.49)	1.29 (1.11, 1.51)
LDL-C, (mmol/L)	1.93 (1.60, 2.24)	2.16 (1.80, 2.59)	2.29 (1.91, 2.64)
ApoA1, (g/L)	1.30 (1.20, 1.41)	1.31 (1.19, 1.42)	1.32 (1.21, 1.44)
ApoB, (g/L)	0.57 (0.05, 0.66)	0.65 (0.54, 0.74)	0.68 (0.58, 0.79)
sdLDLC-C, (mmol/L)	0.38 (0.31, 0.46)	0.43 (0.34, 0.53)	0.45 (0.36, 0.57)

NW, normal weight; OW, overweight; OB, obesity.

### Multivariable Adjusted β and 95% Confidence Interval for Body Mass Index z-Scores Across C1q Tertiles

**Table 2** shows the adjusted *β* and 95% CIs for predicting BMI z-scores across C1q tertiles. In comparison with the lowest tertile group, BMI z-scores were significantly higher in the third C1q tertile (*β*=0.49; 95% CI: 0.35, 0.62; *P* for trend<0.001) after adjustment for age. Adjustment for all of age, gender, ALT, AST, ALP and GGT did not show a change in the correlation between BMI z-scores and C1q (*β*=0.36; 95% CI: 0.23, 0.50; *P* for trend<0.001). There was a statistically significant association between BMI z-scores and C1q in the stratified analysis by the cut-off value of C1q (**Supplementary Table 2**).

**Table 2:** Multivariable adjusted β and 95% confidence interval (CI) for body mass index (BMI) z-scores across C1q tertiles.

	Tertiles, β (95% CI)			
	Tertile1 (n=397)	Tertile2 (n=397)	Tertile3 (n=397)	*P* for trend
Age-adjusted model	0 (Reference)	**0.33 (0.20, 0.47)**	**0.49 (0.35, 0.62)**	**<0.001**
Multiple-adjusted model	0 (Reference)	**0.24 (0.12, 0.37)**	**0.36 (0.23, 0.50)**	**<0.001**

β, regression coefficient; CI, confidence interval; multiple-adjusted model: adjusted for age (in years), sex (boys vs. girls), ALT (U/L), AST (U/L), ALP (U/L), GGT (U/L). P-value for trend was obtained by adjusting tertiles of C1q level as a continuous variable. P-values< 0.05 are in bold.

### Multivariable Adjusted Odds Ratios and 95% Confidence Intervals for Overweight, Obesity, and Metabolic Syndrome Across C1q Tertiles

The associations between C1q and overweight, obesity, and MetS are presented in **Table 3**. It was observed that participants in the highest tertile had a 2.26-fold (95% CI: 1.50, 3.40) higher risk of overweight and obesity than those in the lowest tertile after adjusting for age, gender, ALT, AST, ALP, and GGT. The OR (95% CI) for obesity of the highest tertile was 3.76 (2.08, 6.78) when compared with the lowest tertile after adjustment for the same confounding variables. Participants in the highest tertile were more likely to also have MetS compared to those in the lowest tertile, after adjusting for the same variables (OR =5.43; 95% CI: 2.02, 14.60). Similarly, the prevalence of overweight and obesity, obesity, as well as MetS significantly increased across increasing tertiles of C1q (*P* for trend<0.001; *P* for trend<0.001; *P* for trend=0.001). After classifying C1q according to the cut-off value, C1q was significantly related to obesity and MetS (**Supplementary Table 3**).

**Table 3:** Multivariable adjusted odds ratios and 95% confidence interval (CI) for overweight, obesity, and metabolic syndrome (MetS) across C1q tertiles.

	Tertiles, OR (95% CI)			
	Tertile1 (n=397)	Tertile2 (n=397)	Tertile3 (n=397)	*P* for trend
Overweight+obesity				
Age-adjusted model	1 (Reference)	**2.12 (1.53, 2.96)**	**2.80 (2.02, 3.88)**	**<0.001**
Multiple-adjusted model	1 (Reference)	**1.85 (1.27, 2.68)**	**2.26 (1.50, 3.40)**	**<0.001**
Obesity				
Age-adjusted model	1 (Reference)	**2.35 (1.43, 3.87)**	**3.18 (1.97, 5.15)**	**<0.001**
Multiple-adjusted model	1 (Reference)	**2.35 (1.35, 4.08)**	**3.76 (2.08, 6.78)**	**<0.001**
MetS				
Age-adjusted model	1 (Reference)	**4.38 (1.78, 10.80)**	**5.10 (2.09, 12.43)**	**<0.001**
Multiple-adjusted model	1 (Reference)	**4.38 (1.68, 11.40)**	**5.43 (2.02, 14.60)**	**0.001**

OR, odds ratio; CI, confidence interval; Multiple-adjusted model: adjusted for age (in years), sex (boys vs. girls), ALT (U/L), AST (U/L), ALP (U/L), GGT (U/L). P-value for trend was obtained by adjusting tertiles of C1q level as a continuous variable. P-values< 0.05 are in bold.

### Multivariable Adjusted Odds Ratios and 95% Confidence Intervals for Metabolic Syndrome Components Across C1q Tertiles

The adjusted ORs and 95% CIs for components of MetS across C1q tertiles are reported in **Table 4**. In comparison to subjects in the lowest tertile, those in the highest tertile had a significantly increased risk of central obesity (OR =3.60; 95% CI: 2.42, 5.34; *P* for trend<0.001), high TG (OR =2.33; 95% CI: 1.19, 4.55; *P* for trend=0.010), and low HDL-C (OR =2.31; 95% CI: 1.65, 3.22; *P* for trend<0.001) after adjusting for age, and even after adjustment for relevant confounders, including age, gender, ALT, AST, ALP, and GGT. Although no association between C1q and hypertension was observed after adjusting for age, participants in the highest tertile had a 1.99-fold (95% CI: 1.30, 3.05) higher risk of hypertension than those in the lowest tertile after adjusting for the additional variables described above. No association between C1q and hyperglycemia was reported. After classifying C1q according to the cut-off value, the C1q level was significantly related to components of MetS (except for FPG and high TG) (**Supplementary Table 4**).

**Table 4:** Multivariable adjusted odds ratios and 95% confidence interval (CI) for metabolic syndrome (MetS) components across C1q tertiles.

	Tertiles, OR (95% CI)			
	Tertile1 (n=397)	Tertile2 (n=397)	Tertile3 (n=397)	*P* for trend
Central obesity				
Age-adjusted model	1 (Reference)	**2.31 (1.53, 3.49)**	**3.60 (2.42, 5.34)**	**<0.001**
Multiple-adjusted model	1 (Reference)	**1.88 (1.20, 2.95)**	**2.57 (1.60, 4.13)**	**<0.001**
Hypertension				
Age-adjusted model	1 (Reference)	1.32 (0.94, 1.85)	1.22 (0.87, 1.72)	0.251
Multiple-adjusted model	1 (Reference)	**1.56 (1.08, 2.26)**	**1.99 (1.30, 3.05)**	**0.001**
Hyperglycemia				
Age-adjusted model	1 (Reference)	2.01 (0.37, 11.04)	3.96 (0.83, 18.78)	0.063
Multiple-adjusted model	1 (Reference)	1.45 (0.25, 8.49)	2.19 (0.40, 11.93)	0.328
High TG				
Age-adjusted model	1 (Reference)	1.32 (0.63, 2.76)	**2.33 (1.19, 4.55)**	**0.010**
Multiple-adjusted model	1 (Reference)	1.34 (0.62, 2.89)	**2.87 (1.34, 6.16)**	**0.005**
Low HDL-C				
Age-adjusted model	1 (Reference)	**1.68 (1.20, 2.37)**	**2.31 (1.65, 3.22)**	**<0.001**
Multiple-adjusted model	1 (Reference)	1.42 (0.99, 2.02)	**1.70 (1.17, 2.48)**	**0.006**

OR, odds ratio; CI, confidence interval; multiple-adjusted model: adjusted for age (in years), sex (boys vs. girls), ALT (U/L), AST (U/L), ALP (U/L), GGT (U/L). P-value for trend was obtained by adjusting tertiles of C1q level as a continuous variable. P-values< 0.05 are in bold.

### Receiver Operating Characteristic for Predictive Values of C1q Levels in Detecting Metabolic Syndrome

**Figure 1:**
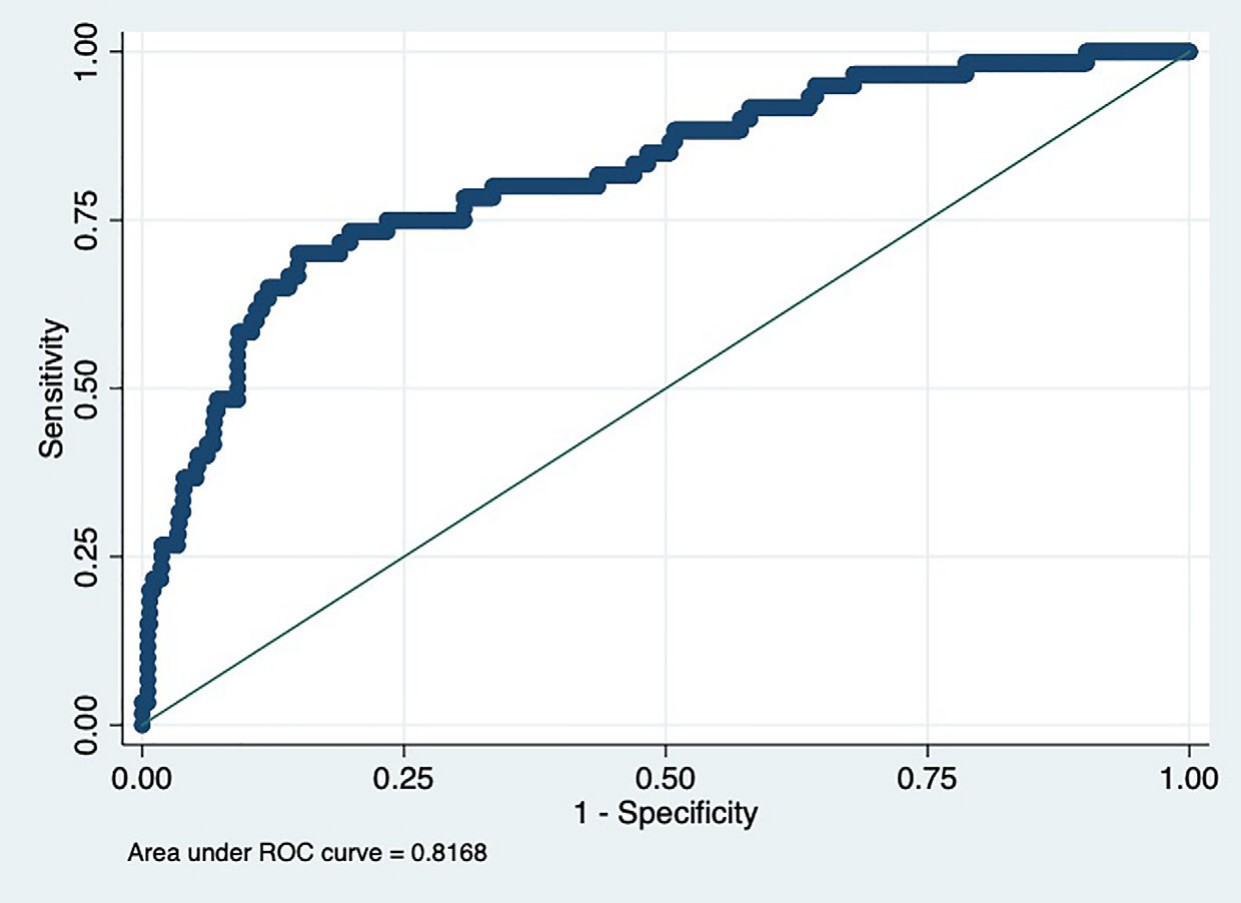
Receiver operating characteristic (ROC) for predictive values of C1q levels in detecting metabolic syndrome (MetS).

The ROC curve for tertiles of C1q in predicting MetS is shown in **Figure 1**. As can be observed, the AUC was 0.82 (95% CI: 0.76, 0.88; *P*<0.001) in the total population after adjusting for age, gender, ALT, AST, ALP, and GGT. According to the cut-off value of C1q, the AUC was 0.83 (95% CI: 0.78, 0.88; *P*<0.001) (**Supplementary Figure 1**).

## Discussion

This was the first study investigating the association between C1q levels and metabolic parameters among adolescents aged 13 to 18 years. In this cross-sectional observational study, we reported a significantly higher prevalence of obesity and MetS features among adolescents with elevated serum C1q levels. The adjustment of potential confounding variables described in the study did not have a substantial impact on the above results. We also found that serum C1q levels were significantly related to components of MetS (except for FPG). Our findings raised the possibility that C1q level may be related to the prediction and prevention of obesity or MetS in adolescents.

As a major part of the innate immune system (38), the complement system was considered to have a critical role in obesity (26). Several studies demonstrated that the complement system is widely expressed and regulates inflammation in adipose tissues (14, 17). Moreover, genes of the classical pathway—the specific complement activation pathway—are widely expressed in human adipose tissue (39). By observing the upregulation of complement genes in adipose tissue of heavier co-twins, C1q A-C genes were upregulated in obesity, and C1q stain was more extensive in obese twins (26). Furthermore, a previous study reported that the expression of C1q increased in epididymal adipose tissue among several models of mice, including genetic mice, high-fat diet-induced obese mice, and Zucker obese rats (40). An observational epidemiological survey in 239 Japanese male subjects reported that serum C1q was positively related to adiposity, such as BMI, WC, visceral fat area, and subcutaneous fat area (41). However, there have been very few studies that have examined the relationship between C1q and obesity in human adolescents. In fact, this study suggested that a positive association exists between serum C1q level and BMI, and there was evidence that higher C1q was significantly related to the prevalence of MetS among adolescents.

Our findings also revealed that serum C1q was positively related to MetS and suggested significant associations of serum C1q with a few components of MetS, including central obesity, hypertension, high TG, and low HDL-C. Nevertheless, there was no significant association between C1q and hyperglycemia in the present study. This might be attributed to a relatively small population of hyperglycemia in comparison to other components of MetS among the study subjects. Previous studies on C1q and MetS as well as its components were in line with our results. In a study of subjects aged 30–74 years, there were clear positive associations between C1q and several factors related to MetS, such as systolic blood pressure, diastolic blood pressure, and log TG; the inverse association between C1q and HDL-C was also reported (41). A study conducted among people of Caucasian descent above 40 years showed significant associations of C1q with TG, HDL-C, and FPG. As reported in that study, the relationship between C1q and MetS was modest (42), which was inconsistent with our results. This difference may be attributed to the subjects belonging to a specific population of individuals who already exhibit moderately increased risk of cardiometabolic diseases. Studies have demonstrated that MetS was associated with chronic inflammatory responses (43, 44). Meanwhile, complement activation by C1q could exacerbate many chronic inflammatory diseases (45). Although the pathophysiology of MetS has yet to be fully explained, insulin resistance has proven to be a critical contributor to MetS (46, 47). The complement system is involved in several aspects of the histopathophysiology that lead to insulin resistance. In the C1q-knocked mouse model, the study demonstrated that C1q had a protective role in insulin resistance induced by a high-fat diet (48).

The strengths of this study were the relatively large sample size of the Chinese adolescent population, the appropriate study design, and the standardized information collection procedures. Moreover, measurement biases were largely avoided since blood samples were analyzed by clinical laboratory standards, which improved the reliability of our study. Despite the noteworthy findings revealed in the present study, it has several limitations. First, the cross-sectional design of the current study might limit the strength of these findings as it is not possible to investigate the longitudinal association between C1q and MetS. Further prospective studies are warranted to establish a cause-effect relationship. Second, subjects with inflammations or infections may have altered C1q levels. However, inflammatory markers such as C-reactive protein were not measured in the current study. Inflammatory markers should be included in future studies. Also, not all potential factors to obesity or MetS were included in the present study. To minimize the effect of potential covariates, we have considered a variety of covariates, such as ALT, AST, ALP, and GGT, but we understand that there are many other possible factors to consider. Finally, the present study was limited to Chinese adolescents, which may not be completely representative of other populations.

## Conclusions

This study demonstrated a significant association between C1q and the prevalence of obesity in a large sample of Chinese adolescents. There were also significant associations between C1q level and metabolic parameters (except for FPG) in Chinese adolescents aged 13 to 18 years. Based on these findings, we suggest that C1q may present as a novel biomarker of obesity and MetS in adolescents. The molecular mechanism of complement C1q activation about MetS needs further investigation.

## Data Availability Statement

The raw data supporting the conclusions of this article will be made available by the authors, without undue reservation.

## Ethics Statement

The study was approved by the China Medical University Health Science Ethics Committee, and was conducted in accordance with the principles of the Declaration of Helsinki. The written informed consent of all the participants and/or their parents has been obtained before entering the study.

## Author Contributions

DW, ZZ, and SZ participated in the study design and organized the data collection. XY, YM, and SZ analyzed and interpreted the data. XY wrote the manuscript. All authors have read and agreed to the final manuscript. All authors contributed to the article and approved the submitted version.

## Funding

This research was supported by the National Natural Science Foundation of China [71774173], CMB Open Competition (CMB-OC), Research Program Health Policy and System Sciences [18-291], and the Liaoning province key research and development plan guidance plan resource platform construction project [2017225004].

## Conflict of Interest

The authors declare that the research was conducted in the absence of any commercial or financial relationships that could be construed as a potential conflict of interest.

## Abbreviations

ALP, Alkaline phosphatase; ALT, Alanine aminotransferase; ApoA1, Apolipoprotein A1; ApoB, Apolipoprotein B; AST, Aspartate aminotransferase; AUC, Area under the curve; BMI, Body mass index; C1q, Complement C1q; CI, Confidence interval; FPG, Fasting plasma glucose; GGT, Gamma glutamyl transpeptidase; HDL-C, High-density lipoprotein cholesterol; IDF, International Diabetes Federation; LDL-C, Low-density lipoprotein cholesterol; MetS, Metabolic syndrome; OR, Odds ratio; ROC, Receiver operating characteristic; sdLDL-C, Small dense low-density lipoprotein cholesterol; TG, Triglyceride; WHO, World Health Organization; WC, Waist circumference; NW, normal weight; OW, overweight; OB, obesity.
