# Supplementary figures and images for: Complement C1q as a Potential Biomarker for Obesity and Metabolic Syndrome in Chinese Adolescents
Source: Front Endocrinol (Lausanne). 2020 Nov 30;11:586440. doi: 10.3389/fendo.2020.586440 (PMC7735390; doi:10.3389/fendo.2020.586440)

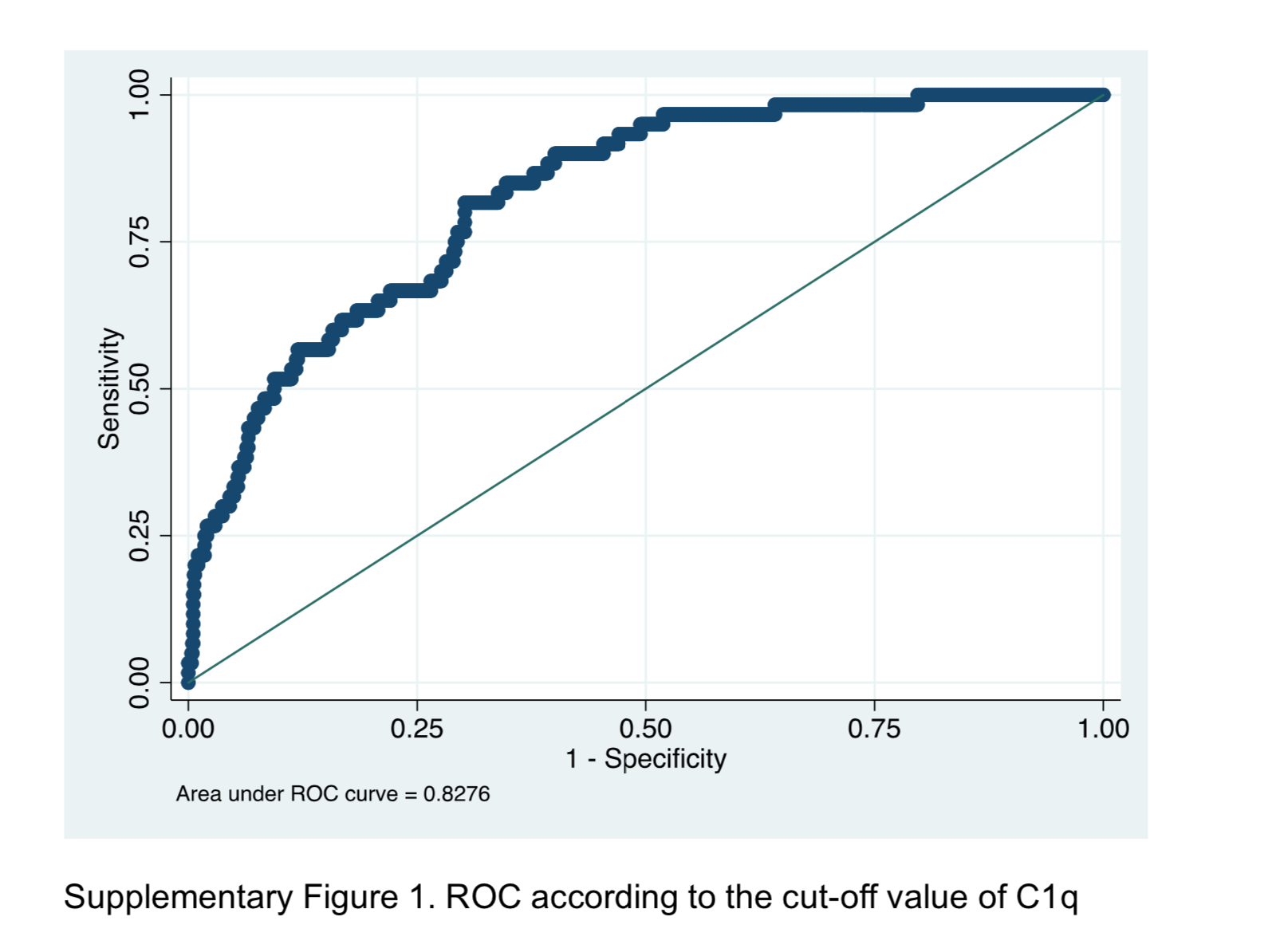

Supplement: Supplementary file 1 [file Image_1.png]
