# Supplementary material for: Complement C1q as a Potential Biomarker for Obesity and Metabolic Syndrome in Chinese Adolescents
Source: Front Endocrinol (Lausanne). 2020 Nov 30;11:586440. doi: 10.3389/fendo.2020.586440 (PMC7735390; doi:10.3389/fendo.2020.586440)
Supplement: Supplementary file 2 [file Table_1.docx]

Supplementary Table 1. Characteristics of the study population according to the cut-off value of C1q

| Characteristic | Lower (n=654) | Upper (n=537) |
| --- | --- | --- |
| Age, mean (SD), years | 16.25 (0.99) | 16.18 (1.00) |
| Boys, No. (%) | 430 (65.75) | 148(27.56) |
| Anthropometry |  |  |
| BMI z-score, mean (SD) | -0.15 (0.86) | 0.25 (1.09) |
| Weight status, No. (%) |  |  |
| NW | 508 (77.68) | 324 (60.34) |
| OW | 90 (13.76) | 119 (22.16) |
| OB | 56 (8.56) | 94 (17.50) |
| Waist Circumference, mean (SD), cm | 73.05 (9.63) | 75.54 (12.08) |
| Metabolic syndrome outcomes, No. (%) |  |  |
| Metabolic syndrome | 16 (2.45) | 44 (8.19) |
| Central obesity | 82 (12.54) | 154 (28.68) |
| Hypertension | 136 (20.80) | 134 (24.95) |
| Hyperglycemia | 3 (0.46) | 11 (2.05) |
| High TG | 26 (3.98) | 33 (6.15) |
| Low HDL-C | 138 (21.10) | 169 (31.47) |
| Laboratory examinations, median (Q1, Q3), |  |  |
| ALT, (U/L) | 26 (11, 62) | 30.5 (12, 62) |
| AST, (U/L) | 16 (14, 18) | 15 (13, 18) |
| ALP, (U/L) | 100 (78, 131) | 89 (74, 111) |
| GGT, (U/L) | 16 (13, 20) | 16 (13, 22) |
| FPG, (mmol/L) | 4.24 (3.94, 4.51) | 4.36 (4.07, 4.65) |
| HDL-C, (mmol/L) | 1.30 (1.14, 1.5) | 1.28 (1.11, 1.49) |
| LDL-C, (mmol/L) | 1.99 (1.67, 2.39) | 2.28 (1.90, 2.64) |
| ApoA1, (g/L) | 1.30 (1.20, 1.42) | 1.31 (1.2, 1.44) |
| ApoB, (g/L) | 0.60 (0.51, 0.69) | 0.67 (0.58, 0.77) |
| sdLDLC-C, (mmol/L) | 0.39 (0.32, 0.50) | 0.45 (0.36, 0.56) |

Note: NW: normal weight; OW: overweight; OB: obesity.
