# Supplementary material for: Complement C1q as a Potential Biomarker for Obesity and Metabolic Syndrome in Chinese Adolescents
Source: Front Endocrinol (Lausanne). 2020 Nov 30;11:586440. doi: 10.3389/fendo.2020.586440 (PMC7735390; doi:10.3389/fendo.2020.586440)
Supplement: Supplementary file 3 [file Table_2.docx]

Supplementary Table 2. Multivariable adjusted β (95% CI) and *P*-value for BMI z-scores according to the cut-off value of C1q

|  | β (95% CI) | *P-*value |
| --- | --- | --- |
| Age-adjusted model | **0.39 (0.28, 0.50)** | **<0.001** |
| Multiple-adjusted model | **0.27 (0.16, 0.38)** | **<0.001** |

Note: β: regression coefficient; CI: confidence interval; Multiple-adjusted model: adjusted for age (in years), sex (boys vs. girls), ALT (U/L), AST (U/L), ALP (U/L), GGT (U/L). *P*-values< 0.05 are in bold.
