# Supplementary material for: Complement C1q as a Potential Biomarker for Obesity and Metabolic Syndrome in Chinese Adolescents
Source: Front Endocrinol (Lausanne). 2020 Nov 30;11:586440. doi: 10.3389/fendo.2020.586440 (PMC7735390; doi:10.3389/fendo.2020.586440)
Supplement: Supplementary file 4 [file Table_3.docx]

Supplementary Table 3. Multivariable adjusted OR (95%CI) and *P*-value for overweight, obesity, and MetS according to the cut-off value of C1q

|  | OR (95% CI) | *P*-value |
| --- | --- | --- |
| Overweight+Obesity |  |  |
| Age-adjusted model | **2.27 (1.76, 2.93)** | **<0.001** |
| Multiple-adjusted model | **1.95 (1.42, 2.68)** | **<0.001** |
| Obesity |  |  |
| Age-adjusted model | **2.24 (1.57, 3.19)** | **<0.001** |
| Multiple-adjusted model | **2.30 (1.48, 3.58)** | **<0.001** |
| MetS |  |  |
| Age-adjusted model | **3.54 (1.97, 6.35)** | **<0.001** |
| Multiple-adjusted model | **3.41 (1.75, 6.65)** | **<0.001** |

Note: OR: Odds ratio; CI: confidence interval; Multiple-adjusted model: adjusted for age (in years), sex (boys vs. girls), ALT (U/L), AST (U/L), ALP (U/L), GGT (U/L). *P*-values< 0.05 are in bold.
