# Supplementary material for: Complement C1q as a Potential Biomarker for Obesity and Metabolic Syndrome in Chinese Adolescents
Source: Front Endocrinol (Lausanne). 2020 Nov 30;11:586440. doi: 10.3389/fendo.2020.586440 (PMC7735390; doi:10.3389/fendo.2020.586440)
Supplement: Supplementary file 5 [file Table_4.docx]

Supplementary Table 4. Multivariable adjusted OR (95%CI) and *P*-value for MetS components according to the cut-off value of C1q

|  | OR (95% CI) | *P*-value |
| --- | --- | --- |
| Central obesity |  |  |
| Age-adjusted model | **2.79 (2.06, 3.76)** | **<0.001** |
| Multiple-adjusted model | **2.15 (1.50, 3.09)** | **<0.001** |
| Hypertension |  |  |
| Age-adjusted model | 1.26 (0.96, 1.65) | 0.095 |
| Multiple-adjusted model | **1.77 (1.26, 2.49)** | **0.001** |
| Hyperglycemia |  |  |
| Age-adjusted model | **4.45 (1.23, 16.05)** | **0.023** |
| Multiple-adjusted model | 3.04 (0.75, 12.27) | 0.118 |
| High TG |  |  |
| Age-adjusted model | 1.58 (0.93, 2.68) | 0.088 |
| Multiple-adjusted model | 1.55 (0.84, 2.86) | 0.165 |
| Low HDL-C |  |  |
| Age-adjusted model | **1.76 (1.35, 2.29)** | **<0.001** |
| Multiple-adjusted model | **1.37 (1.02, 1.85)** | **0.035** |

Note: OR: Odds ratio; CI: confidence interval; Multiple-adjusted model: adjusted for age (in years), sex (boys vs. girls), ALT (U/L), AST (U/L), ALP (U/L), GGT (U/L). *P*-values< 0.05 are in bold.
